# Neutrophil phenotypes implicated in the pathophysiology of post-traumatic sepsis

**DOI:** 10.3389/fmed.2022.982399

**Published:** 2022-12-02

**Authors:** Asumi Mizugaki, Takeshi Wada, Takumi Tsuchida, Yoshitaka Oda, Katsuhide Kayano, Kazuma Yamakawa, Shinya Tanaka

**Affiliations:** ^1^Division of Acute and Critical Care Medicine, Department of Anesthesiology and Critical Care Medicine, Faculty of Medicine, Hokkaido University, Sapporo, Japan; ^2^Department of Cancer Pathology, Faculty of Medicine, Hokkaido University, Sapporo, Japan; ^3^Department of Emergency Medicine, Osaka Medical and Pharmaceutical University, Takatsuki, Japan; ^4^Institute for Chemical Reaction Design and Discovery (WPI-ICReDD), Hokkaido University, Sapporo, Japan

**Keywords:** neutrophil phenotype, post-traumatic sepsis, innate immune system, CD11b, SIRPα, Siglec-F, CD68

## Abstract

**Background:**

The disruption of immune homeostasis after trauma is a major cause of post-traumatic organ dysfunction and/or sepsis. Recently, a variety of neutrophil phenotypes with distinct functions have been identified and suggested as involved in various clinical conditions. The association between neutrophil phenotypes and post-traumatic immunodeficiency has also been reported, yet the specific neutrophil phenotypes and their functional significance in post-traumatic sepsis have not been fully clarified. Therefore, we sought to investigate neutrophil phenotypic changes in a murine model, as these may hold prognostic value in post-traumatic sepsis.

**Materials and methods:**

Third-degree burns affecting 25% of the body surface area were used to establish trauma model, and sepsis was induced 24 h later through cecal ligation and puncture (CLP). The Burn/CLP post-traumatic sepsis model and the Sham/CLP control model were established to assess the immunological status after trauma. Histopathological evaluation was performed on the spleen, liver, kidneys, and lung tissues. Immunological evaluation included the assessment of neutrophil markers using mass cytometry as well as cytokine measurements in serum and ascitic fluid through multiplex analysis using LUMINEX^®^.

**Results:**

The Burn/CLP group had a lower survival rate than the Sham/CLP group. Histopathological examination revealed an impaired immune response and more advanced organ damage in the Burn/CLP group. Furthermore, the Burn/CLP group exhibited higher levels of transforming growth factor-beta 1 in the blood and generally lower levels of cytokines than the Sham/CLP group. CD11b, which is involved in neutrophil adhesion and migration, was highly expressed on neutrophils in the Burn/CLP group. The expression of CD172a, which is related to the inhibition of phagocytosis, was also upregulated on neutrophils in the Burn/CLP group. The expression of sialic acid-binding lg-like lectin F and CD68 also differed between the two groups.

**Conclusion:**

Different neutrophil phenotypes were observed between Burn/CLP and Sham/CLP groups, suggesting that neutrophils are implicated in the immune imbalance following trauma. However, further studies are needed to prove the causal relationships between neutrophil phenotypes and outcomes, including survival rate and organ dysfunction.

## Introduction

For over 50 years, trauma has been one of the leading causes of death worldwide, especially among working-age populations (1, 2). Although many patients die due to macrovascular injuries, severe brain injury, or hemorrhage in the hyperacute or acute phase after trauma, survival rates have increased owing to the improved management of trauma patients through transfusion therapy and surgical hemostasis techniques, among other medical advances (3–5).

Despite surviving the acute phase of trauma, a number of patients lose their lives within days to weeks after injury (6, 7). Subacute death in trauma patients is associated with the disruption of immune homeostasis (8). Once trauma occurs, the innate immune system drives a pro-inflammatory response, and an anti-inflammatory response mediated by the adaptive immune system then arises in response to an excessive initial pro-inflammatory signaling (8). An imbalance between pro- and anti-inflammatory responses can lead to the development of systemic inflammatory response syndrome (SIRS) or compensatory anti-inflammatory response syndrome (CARS), resulting in organ damage and/or sepsis (7, 8). However, the treatment of post-traumatic conditions caused by an immune imbalance mainly consists of supportive care, and no curative treatment has yet been established.

The innate immune response eliminates invading pathogens at the initial stage of infection, mainly through the activity of neutrophil and monocyte lineages (9, 10). In recent years, the existence of neutrophil phenotypes with distinct functions has been recognized (11–14). In particular, anti-inflammatory neutrophils have been suggested as involved in the development of CARS and subsequent secondary infections (9, 14). Polymorphonuclear myeloid-derived suppressor cells, which are produced by a variety of infections, including bacterial, and known to act as immunosuppressive agents in sepsis, have also attracted attention in recent years (15). Moreover, neutrophils polarize in the same manner as pro-inflammatory and anti-inflammatory macrophages, which are activated by Th1 and Th2 type cytokines, respectively (16). In the field of cancer, it has been shown that inhibition of transforming growth factor-beta 1 (TGF-β1) signaling, which is implicated in cell death and differentiation, increases the number of pro-inflammatory neutrophils (16). A previous study showed that the balance between pro- and anti-inflammatory neutrophils may be involved in the ventricular remodeling process during myocardial inflammation after infarction (17). Further, toll-like receptor (TLR) 4 deficiency in the central nervous system protected the brain after stroke by increasing the percentage of anti-inflammatory neutrophils (18).

Immunodeficiency after trauma has been studied from various perspectives (6–8, 19–24), including through a focus on neutrophils (25–27). However, the involvement of neutrophils in the pathogenesis of post-traumatic sepsis remains elusive. To investigate this issue, we conducted a study with two features. First, we employed the burn injury model as the experimental trauma model in this study. This model causes tissue damage with decreased circulating blood and has been used in many post-traumatic immunological studies (6, 7, 20–24). By using this model, we can avoid traumatic brain injury, which leads to specific immune responses (28, 29). Moreover, unlike other trauma models, the third-degree burn model is favorable from the perspective that there is no pain after the injury. Second, we employed mass cytometry (cytometry by time-of-flight: CyTOF) to reveal a wide range of functional changes in neutrophils and to investigate neutrophil phenotypes, which may influence the prognosis of post-traumatic sepsis patients.

## Materials and methods

### Ethics statement

All animal protocols performed in this study were approved by the Hokkaido University Animal Experiment Regulations and the Institutional Ethical Review Board at Hokkaido University (Approval number: 19-0167).

### Mice

Outbred male Institute of Cancer Research (ICR) mice were purchased from the Sankyo Labo Service Corporation, Inc. (Tokyo, Japan). Mouse housing was set up to maintain the appropriate temperature and humidity, with automatic light/dark switching every 12 h. The mice were supplied with standard chow (Nosan Corporation, Yokohama, Japan) and water *ad libitum* for the entire study. The mice were acclimated to the environment for at least 72 h before use in the experiments.

### Trauma model

A burn trauma model was established as previously described (20, 22–24, 30). The mice were anesthetized *via* intraperitoneal administration of ketamine/xylazine (125/20 mg/kg), and buprenorphine (0.05 mg/kg) was subcutaneously administered for analgesia before treatment. After shaving of the dorsal hair, an anesthetized mouse was placed and immobilized in a plastic mold so as to expose 25% of the body surface area. The exposed dorsum was immersed in 90°C hot water for 9 s to induce burn, and in 24°C water for 9 s for the sham model. A subcutaneous injection of 1 ml of 0.9% pyrogen-free saline was administered immediately after the resuscitation procedure. A full-thickness and well-demarcated anesthetic third-degree burn injury was induced. No post-injury analgesics were used because the burns were applied to a painless depth. The mortality rate of model mice established using this protocol was below 5%, as previously reported (6, 7, 20, 23, 24) and observed in our laboratory.

### Sepsis model

A cecal ligation and puncture (CLP) sepsis model was established as previously described (31–33). The mice were anesthetized in the same manner as for the burn protocol, and their abdominal fur was shaved. A 1 cm skin and peritoneal incision was made vertically in the midline of the abdomen, and the cecum was gently exposed after resection of the adipose tissue on both sides (34). Ligation was performed at 50% of the cecum, with two punctures with a 21-gauge needle 5 mm from the blind end. The method yielded a survival rate equivalent to the sepsis survival rate in humans (65–80%) (35–37). The cecum was returned to the abdominal cavity, whereafter the peritoneum was sutured with five stitches and the skin with three stitches using a 4-0 nylon thread. Resuscitation was performed *via* subcutaneous injection of 1 ml 0.9% pyrogen-free saline after CLP. The CLP procedure was performed on a hot plate (Heater Mat KN-475, Natsume Seisakusho, Co., Ltd., Tokyo, Japan) and maintained at approximately 38°C. To prevent surgical site infection, post-operative mice were kept in the supine position until the anesthetic effect wore off and they regained consciousness. To prevent post-operative hypothermia, the cage was placed on a hot plate until the mice woke up from anesthesia (38). Antibiotic treatment began 2 h after the CLP procedure, with a subcutaneous injection of imipenem/cilastatin (25 mg/kg) repeated every 12 h for the first 3 days (for a total of six injections) (39).

### Schedule of experiments

Two murine models of post-traumatic sepsis, the Burn/CLP and Sham/CLP models, were established. The first day of trauma infliction was set as day 0, and CLP was performed 24 h after injury (day 1). Fifteen mice from each group were followed up until day 15 (14 days after CLP) to determine survival rates. Five mice from each group were used for histopathological, immunological, and serological evaluation. For histopathology only, five mice with neither trauma nor sepsis were used as controls in addition to the Burn/CLP and Sham/CLP models. Samples for histopathological, immunological, and serological examination were collected 24 h after CLP.

### Histopathological evaluation

Twenty-four h after CLP, the mice were anesthetized and euthanized through the intraperitoneal administration of ketamine/xylazine (250/40 mg/kg) at twice the dose used in trauma and sepsis models, and were then placed in a supine position. The liver, kidney, spleen, and lung tissues were excised from five mice per group for histological evaluation. The organs were fixed in 10% formalin neutral buffer solution (FUJIFILM Wako Pure Chemical Corporation, Osaka, Japan) for 2 weeks, following the fixation method in our other study, and the slides were prepared by Morphotechnology Co., Ltd. (Sapporo, Japan). Hematoxylin and eosin (H&E) staining was performed.

### Cell preparation

Ascitic fluid and blood samples were collected from five mice per group. The mice were anesthetized and euthanized as done for histopathological evaluation and were then placed in a supine position. The abdominal skin was newly incised avoiding the CLP wound, the exposed peritoneum was then punctured, and 5 ml of 0.9% pyrogen-free saline was injected into the peritoneal cavity. The abdomen of each mouse was rubbed gently to ensure that the injected saline was evenly mixed. A 5 mm peritoneal incision was then made, through which as much ascitic fluid as possible (3–4 ml) was collected using a 2.5 ml syringe. Ascitic fluid was collected in a centrifuge tube pre-filled with 0.5 ml of 169 mM EDTA (EDTA tripotassium salt dihydrate 0.075 g/ml of water). Terminal blood collection was subsequently conducted on mice maintained under anesthesia by puncturing the cardiac fossa with a 1 ml syringe pre-filled with 0.1 ml of 169 mM EDTA.

Cells in the samples were prepared in culture medium (C5) containing RPMI 1640, 5% heat-inactivated FCS, 1 mM glutamine, 10 mM HEPES, 2 mM non-essential amino acids, penicillin/streptomycin/fungizone, and 2.5 × 10^–5^ M 2-mercaptoethanol. All of the above-listed products included in the C5 medium were purchased from Life Technologies Corporation (Carlsbad, CA, USA). Blood samples were washed and centrifuged repeatedly with C5 after removal of red blood cells (RBCs) using ammonium chloride-based mouse RBC lysis buffer (JL-buffer), which was developed by the Lederer Lab at Harvard Medical School (20). Ascitic fluid samples were also washed and centrifuged repeatedly using C5. The pellet containing cells at the bottom of the centrifuge tube was collected with 0.5 ml of cell freezing medium CryoStor^®^ (Biolife Solutions, Inc., Bothell, WA, USA) and stored in a tube. To avoid rapid cooling, the tubes filled with cells in the CryoStor^®^ were set in Mr. Frosty^®^ (Thermo Fisher Scientific, Waltham, MA, USA), which had been pre-chilled in a refrigerator at 4°C. The tubes with cells in Mr. Frosty were first placed in a refrigerator at 4°C for 20 min and then kept in a −80°C freezer overnight (12–24 h). The tubes with cells were taken from Mr. Frosty and immediately stored at −196°C in a liquid nitrogen tank until mass cytometry was performed.

### Mass cytometry

Cytometry by time-of-flight was performed for immunological evaluation. All CyTOF staining procedures were performed at room temperature. Cells were first stained with a cisplatin viability staining reagent (Fluidigm Sciences, South San Francisco, CA, USA) for 5 min and washed *via* centrifugation. Fc-blocking agents were added to the cells for 10 min before adding the CyTOF antibody staining cocktail. Cells were stained for 30 min and washed with CyTOF staining buffer (calcium/magnesium-free PBS, 0.05% sodium azide, and 0.2% bovine serum albumin). For fixation and permeabilization of the cells, we used Maxpar^®^ Fix I Buffer/Maxpar 10X Barcode Perm Buffer (Fluidigm Sciences), which was incubated with a palladium-based barcode reagent for 30 min. The samples were pooled into a single tube after washing off the barcode reagent. The Maxpar Nuclear Antigen Staining Buffer Set (Fluidigm Sciences) was used to fix and permeabilize the cells, which were then incubated with an intracellular antibody cocktail. After addition of the intracellular antibody cocktail for 30 min, the cells were washed and fixed with 1.6% paraformaldehyde. The cells and iridium intercalator solution (Max-Par Intercalator-Ir 500 mM; Fluidigm Sciences) were added together for 20 min, followed by a final wash. Cells were incubated with MilliQ-filtered distilled water (EMD Millipore, Billerica, MA, USA) at a concentration of 1 × 10^6^ cells/ml containing EQ calibration beads (EQ Four Element Calibration Beads; Fluidigm Sciences). The cells were analyzed using a Helios mass cytometer (Fluidigm Sciences) on pooled single samples using the Normalizer and Single Cell Debarker software developed at the Nolan Lab (Stanford, Palo Alto, CA, USA). Normalization and deconvolution were also performed. The CyTOF staining panels used in this study are listed in Supplementary Tables 1, 2. OMIQ (Omiq, Inc., Santa Clara, CA, USA) was used for mass cytometric analysis. In OMIQ, we used automated optimized parameters for T-distributed stochastic neighbor embedding (opt-SNE) for the dimension reduction algorithm and FlowSOM for the clustering and visualization algorithms.

### Serological evaluation

Cytokines in the serum and ascitic fluid were evaluated by multiplex analysis using Luminex^®^ 100/200™. Five mice from each group were used for evaluation. Blood and ascitic fluid were collected 24 h after CLP in the same manner as the samples for mass cytometry. The obtained blood samples were centrifuged at 700 × *g* for 20 min at 25°C, and the ascitic fluid samples were centrifuged at 200 × *g* for 5 min at 25°C. The supernatants were collected and stored in a freezer at −20°C. The samples were subjected to multiplex analysis by Luminex^®^ 100/200™ using the MILLIPLEX^®^ MAP kit (Merck Millipore Corporation, Darmstadt, Germany).

### Statistical analysis

Statistical analyses and calculations were performed using GraphPad Prism for Windows version 9.3.1 (Graph Pad Software, San Diego, CA, USA). Survival curves were estimated *via* the Kaplan–Meier method, and differences in survival were evaluated using the log-rank test. Numerical data from the OMIQ were exported and compared among groups using the two-sided non-parametric Mann–Whitney *U* test to assess significance. Statistical significance was set at *p* < 0.05.

## Results

### Survival outcome after cecal ligation and puncture

**FIGURE 1:**
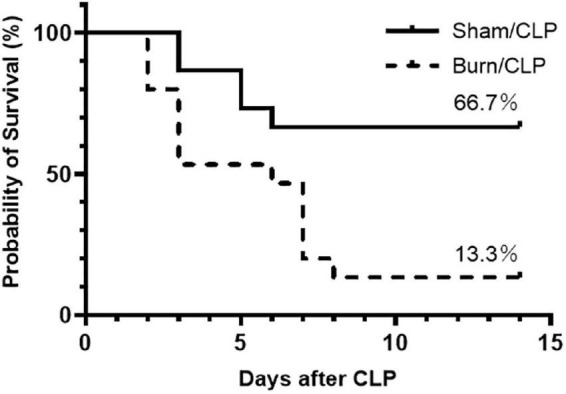
Kaplan–Meier survival curve for 14-day survival after CLP. CLP, cecal ligation and puncture.

The 14-day survival rate after CLP was compared between the Burn/CLP and Sham/CLP groups, using 15 mice per group. A significant reduction in survival was observed in the Burn/CLP group (13.3%) when compared to the Sham/CLP group (66.7%) (log-rank, *p* = 0.006) (Figure 1).

### Histopathological findings of major organs

**FIGURE 2:**
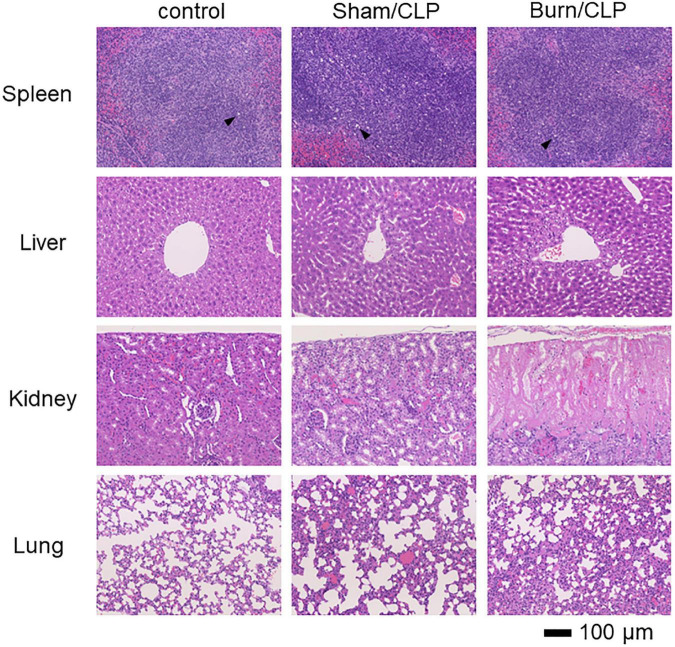
Histopathological findings in the Burn/CLP group, Sham/CLP group, and control cases. 10× (Objective lens) spleen, liver, kidney, and lung tissues. CLP, cecal ligation and puncture.

We performed histopathological analysis of the spleen, liver, kidney, and lung tissues using H&E staining, which did not show staining defects caused by overfixation (Figure 2). Considerable numbers of infiltrating macrophages were observed in the spleens of the Sham/CLP group compared to a few in the Burn/CLP and control groups. The extent of macrophage infiltration into the spleen, scored using the scoring system (Supplementary Figure 1) for control, Sham/CLP, and Burn/CLP mice are shown in Supplementary Figures 2–4. The Sham/CLP group exhibited dilation of the sinusoid, while livers of the Burn/CLP group exhibited hepatocyte damage around the central vein, with dilation of the sinusoid. In the kidneys, acute tubular necrosis was observed in three of the five cases from the Burn/CLP group, while none was observed in the control and the Sham/CLP groups. Congestion was more noticeable in the lungs of the Burn/CLP group than the Sham/CLP group.

### Cytokine levels in serum and ascitic fluid

**FIGURE 3:**
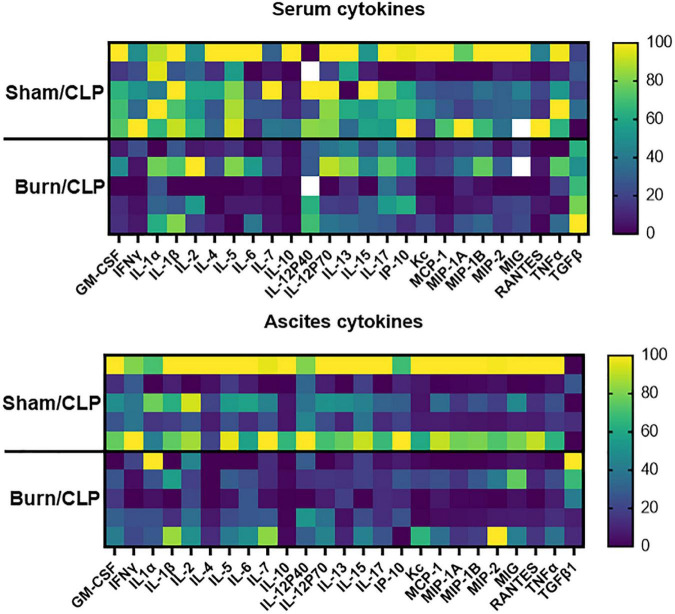
Heatmaps of the cytokine levels in serum and ascitic fluid. CLP, cecal ligation and puncture; GM-CSF, granulocyte-macrophage colony-stimulating factor; IFNγ, interferon-gamma; IL, interleukin; IP, interferon gamma-induced protein; KC, keratinocyte-derived chemokines; MCP, monocyte chemotactic protein; MIG, monokine induced by interferon-gamma; MIP, macrophage inflammatory protein; RANTES, regulated on activation, normal T cell expressed and secreted; TNF, tumor necrosis factor; TGF-β1, transforming growth factor-beta 1.

**FIGURE 4:**
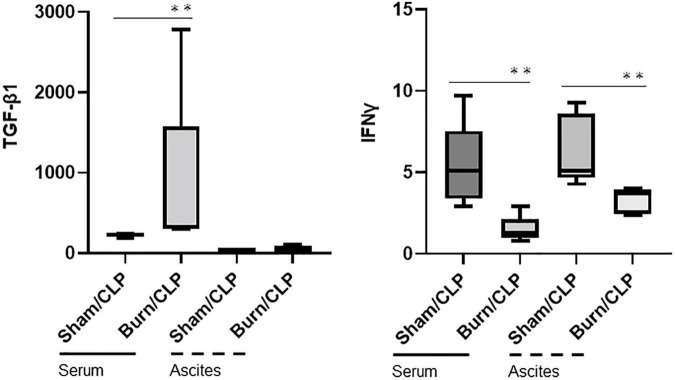
Transforming growth factor-beta 1 and IFNγ levels in serum and ascitic fluid. CLP, cecal ligation and puncture; TGF-β1, transforming growth factor-beta 1; IFN-γ, interferon gamma. ^**^p < 0.01.

The cytokine levels in serum and ascitic fluid are presented in heatmaps (Figure 3). Cytokine levels, including those of interferon-gamma (IFNγ), were generally lower in the Burn/CLP group than in the Sham/CLP group, except for TGF-β1. Serum TGFβ-1 levels were elevated in the Burn/CLP group (Figure 4).

### Cell phenotyping *via* mass cytometry

**FIGURE 5:**
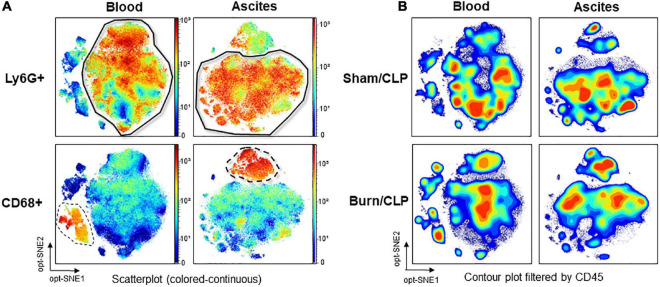
Identification of neutrophils and monocytes/macrophages on the dimension reduction map and CD45 expression. **(A)** Identification of neutrophil islands based on Ly6G and monocyte/macrophage islands based on CD68 in the scatterplot (colored-continuous) of the dimension reduction map, all files concatenated, with the z-channel set to Ly6G or CD68. **(B)** The expression of CD45 in contour plot, with the files concatenated by groups. CD, cluster of differentiation; CLP, cecal ligation and puncture; Ly6G, lymphocyte antigen 6 G; opt-SNE, optimized parameters for T-distributed stochastic neighbor embedding.

The number of events in both groups was adjusted *via* the subsampling method in OMIQ analysis so as to ensure that event numbers are comparable. Lymphocyte antigen 6 G (Ly6G)-positive cells were identified as neutrophils, and CD68-positive cells were identified as monocytes or macrophages through dimension reduction maps created by opt-SNE (Figure 5A). The files were concatenated per group for each blood and ascitic fluid sample, filtered based on CD45, and the contour plots of dimension reduction maps were compared (Figure 5B). CD45-positive cells were differentially abundant between the two groups both in blood and ascitic fluid, with a relative decrease of neutrophil islands in Burn/CLP group.

### Neutrophil marker expression

**FIGURE 6:**
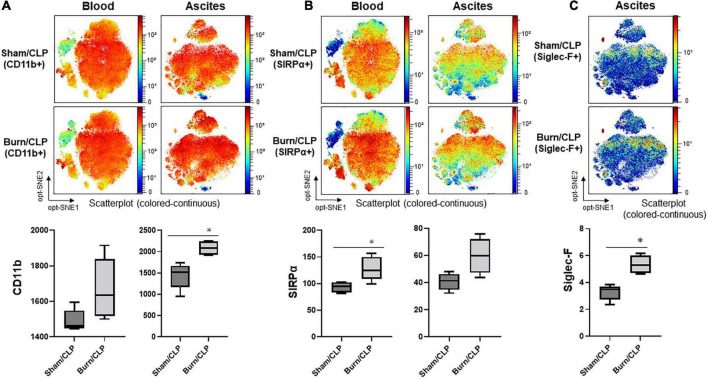
The expression of neutrophil markers. **(A)** The expression of CD11b. **(B)** The expression of SIRPα. **(C)** The expression of Siglec-F. CD, cluster of differentiation; CLP, cecal ligation and puncture; opt-SNE, optimized parameters for T-distributed stochastic neighbor embedding; Siglec-F, sialic acid-binding lg-like lectin F; SIRPα, signal-regulatory protein alpha. *p < 0.05.

**FIGURE 7:**
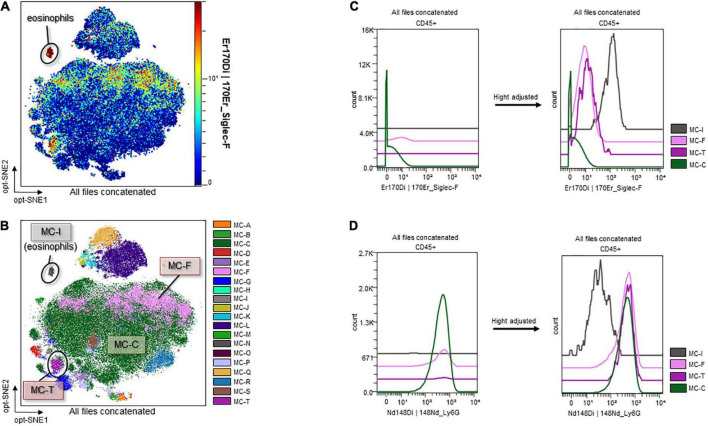
Sialic acid-binding lg-like lectin F and Ly6G expression on eosinophils and Siglec-F-positive/negative neutrophils. **(A)** Identification of eosinophils. **(B)** Metaclusters re-created with emphasis on Siglec-F expression. MC-I: eosinophils; MC-F and MC-T: Siglec-F-positive neutrophils; MC-C: Siglec-F-negative neutrophils. **(C)** Histograms of Siglec-F expression. **(D)** Histograms of Ly6G expression. CD, cluster of differentiation; Ly6G, lymphocyte antigen 6 G; MC, metacluster; opt-SNE, optimized parameters for T-distributed stochastic neighbor embedding; Siglec-F, sialic acid-binding lg-like lectin F.

Marker expression levels are presented *via* heatmaps in Supplementary Figures 5, 6. We examined whether there were any apparent differences in the scatter plot of dimension reduction maps, which were concatenated per group, with the z-channel set to several markers. The expression of CD11b on neutrophils in ascitic fluid was significantly higher in the Burn/CLP group than in the Sham/CLP group. CD11b expression on neutrophils in the blood also tended to be higher in the Burn/CLP group (Figure 6A). CD172a (signal-regulatory protein alpha: SIRPα) appeared as differentially expressed on neutrophils between the two groups, being significantly higher on neutrophils in blood samples of the Burn/CLP group, with a similar trend observed for ascitic fluid samples (Figure 6B). Sialic acid-binding lg-like lectin F (Siglec-F), which is used as an eosinophil surface marker, was partly expressed on the neutrophil island in ascitic fluid samples. The expression of Siglec-F on neutrophils in ascitic fluid exhibited significant differences between the two groups (Figure 6C). Eosinophils, which are generally known to express high levels of Siglec-F, were identified (Figure 7A), and we generated metaclusters including eosinophils (metacluster I: MC-I in Figure 7B), Siglec-F positive neutrophils (MC-F and MC-T in Figure 7B), and Siglec-F negative neutrophils (MC-C in Figure 7B). Siglec-F expression in MC-I, MC-F, MC-T, and MC-C were compared in histograms (Figure 7C). Siglec-F expression in MC-F and MC-T was similar and lower than that in MC-I, i.e., eosinophils. Siglec-F expression in MC-C was even lower than in MC-F and MC-T. In addition, histograms of Ly6G expression in MC-I, MC-F, MC-T, and MC-C (Figure 7D) are shown to reveal that MC-I lacked Ly6G expression while its expression was similar in MC-F, MC-T, and MC-C. Since Siglec-F expression on neutrophils is found mainly in inflammatory tissues and not in peripheral blood, Siglec-F was not included in the blood panel in this study due to conflicts with other markers. We also examined the expression of the above-mentioned markers on monocytes and macrophages, obtaining similar results (Supplementary Figure 7).

### CD68-high neutrophils in the Burn/cecal ligation and puncture group

**FIGURE 8:**
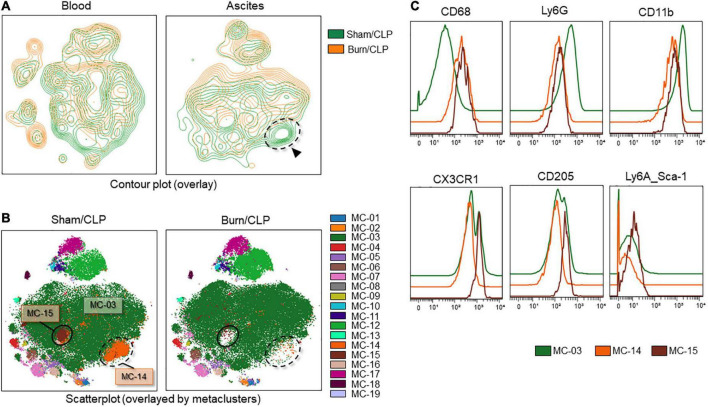
Deficiency of CD68-high neutrophils in the Burn/CLP group. **(A)** Comparison between the Burn/CLP and Sham/CLP groups by contour plot of the dimension reduction map. **(B)** Clustering by FlowSOM. **(C)** Height-adjusted histograms showing differences among the three metaclusters (MC-03, MC-14, and MC-15). CD, cluster of differentiation; CLP, cecal ligation and puncture; CX3CR1, C-X-3C chemokine receptor 1; Ly6, lymphocyte antigen 6; MC, metacluster.

**FIGURE 9:**
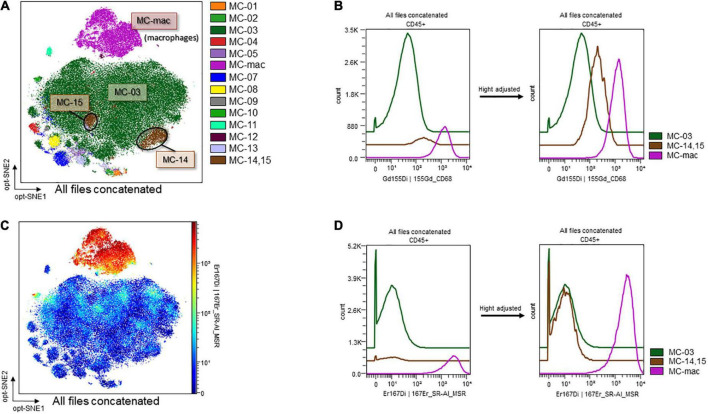
CD68 and MSR expression on macrophages and CD68-high/low neutrophils. **(A)** Metaclusters re-created with emphasis on CD68 expression. MC06: macrophages; MC14 and MC15: CD68-positive neutrophils; MC03: CD68-negative neutrophils. **(B)** MSR expression on an opt-SNE map. **(C)** Histograms of CD68 expression. **(D)** Histograms of MSR expression. CD, cluster of differentiation; MC, metacluster; MSR, macrophage scavenger receptor; opt-SNE, optimized parameters for T-distributed stochastic neighbor embedding.

The contour plots of the two groups were overlaid to assess differences. While notable differences were not recorded for the blood samples, areas without overlap in the neutrophil island were noted for the ascitic fluid samples (Figure 8A, surrounded by the dashed line). The clustering algorithm FlowSOM was used to create metaclusters and examine the non-overlapping area in greater detail. The scatter plot of the dimension reduction map with metaclusters was created. The non-overlapping area is shown as MC-14 (surrounded by the dashed line in Figure 8B) in the metacluster scatter plot. The scatter plot revealed the existence of another non-overlapping metacluster (MC-15, surrounded by the solid line in Figure 8B) on the neutrophil island, which was not apparent in the contour plot when the two groups were overlaid. These two metaclusters, MC-14 and MC-15, which did not overlap between the two groups, were found to be present in the Sham/CLP group but absent in the Burn/CLP group. To characterize both metaclusters, histograms of each cell surface marker were generated, and each metacluster was found to express high levels of CD68 (Figure 8C) when compared to the surrounding largest neutrophil metacluster (shown as MC-03 in Figure 8B). They also exhibited slightly lower CD11b and Ly6G expression when compared to MC-03 cells. Further, MC-15 showed higher expression of C-X-3C chemokine receptor 1 (CX3CR1), CD205, and Ly6A (stem cell antigen-1: Sca-1) than MC-03 or MC-14 cells. We also generated another metacluster map to compare the abundance of CD68 expressions between macrophages (MC-mac in Figure 9A), CD68 positive neutrophils (MC-14 and MC-15 in Figure 9A), and CD68 negative neutrophils (MC-03 in Figure 9A). In Figure 9B, histograms show that MC-mac, MC-14, and MC-15 had higher expression of CD68 when compared to MC-03, but MC14 and MC15 had lower CD68 expression compared to MC-mac which indicates a macrophage island. To confirm that MC-14 and MC-15 are indeed neutrophils and not macrophages, a scatterplot with the Z-channel set to macrophage scavenger receptor (MSR) (Figure 9C) and a histogram showing MSR expression (Figure 9D) are demonstrated. MC-mac had high expression of MSR, while MC-14, MC-15, and MC-03 lacked MSR expression.

## Discussion

In this study, we investigated neutrophil phenotypes implicated in the pathogenesis of post-traumatic sepsis. Histopathological analysis revealed a suppression of the immune response and progressive organ ischemia in the Burn/CLP group, which were consistent with the lower survival rate. We found that the Burn/CLP group exhibited differences in the expression of CD11b, SIRPα, Siglec-F, and CD68 in neutrophils compared to the Sham/CLP group. These phenotypic differences in neutrophils may contribute to the dysregulation of immune homeostasis during post-traumatic sepsis, thus contributing to increased organ damage and reduced survival rates.

The decreased numbers of macrophages in the spleen suggest attenuated immune responses in the Burn/CLP compared to Sham/CLP groups. Hepatocyte damage around the central hepatic vein, acute tubular necrosis in the kidneys, and lung congestion were indicative of more advanced organ ischemia and greater tissue damage in the Burn/CLP group when compared with the Sham/CLP group. These observations were consistent with the decreased survival rate and overall lower cytokine release in the Burn/CLP group. The higher TGF-β1 levels detected in the blood of the Burn/CLP group could be attributed to its role in wound healing (40). TGF-β1 is also known to play an immunosuppressive role by inhibiting cell proliferation and suppressing cytokine release (41), and may therefore contribute to the suppressed immune response observed following trauma. In our study, both pro- and anti-inflammatory cytokines were generally suppressed in the Burn/CLP group. Pro-inflammatory cytokines, such as IFNγ, which are normally released in response to CLP-induced sepsis, may have been suppressed by TGF-β1, resulting in an impaired immune response and undesirable outcome. Figure 3 shows the variation in each mouse. This result is expected because outbred mice, which can have uneven immune responses, were used to generate clinically useful evidence. Furthermore, it is possible that differences in the size of the appendix and the amount of feces in the intestinal tract of each mouse may have influenced the results in the process of creating the CLP-induced sepsis model used in this study. However, we believe it is meaningful that there were clear differences between the two groups despite such variations.

Neutrophils are among the key cellular components of the innate immune system and are mobilized during the earliest stages of infection to directly eliminate target microorganisms through phagocytosis, the production of reactive oxygen species (ROS), the generation of neutrophil extracellular traps (NETs), and apoptosis (9, 42). Furthermore, neutrophils are known to have a significant impact on subsequent adaptive responses through the production of specific cytokines (25, 26). In our study, CD11b expression on neutrophils in both blood and ascitic fluid was higher in the Burn/CLP group, as previously reported for CD11b expression after trauma (9, 43). CD11b plays an important role in the adhesion and migration of neutrophils by forming integrins together with CD18. A previous report has demonstrated that CD11b/CD18 activation by integrin agonists improves inflammatory nephritis *via* enhancement of leukocyte adhesion and suppression of leukocyte migration to the tissues (44). Trauma-induced increased expression of CD11b may lead to the systemic attenuation of neutrophil migration, contributing to poorer outcomes in the Burn/CLP groups compared to the Sham/CLP groups, while this does not indicate a causal relationship.

Signal-regulatory protein alpha, which has recently attracted attention as a candidate target of immune checkpoint inhibitors in the field of cancer (45–48), was highly expressed on neutrophils, monocytes, and macrophages in the Burn/CLP group. SIRPα is a transmembrane protein that is expressed on the surface of phagocytic cells. When CD47 on target cells bind to SIRPα on phagocytes, their phagocytic activity is suppressed as a result of the generated “don’t eat me” signal (47). Therefore, the high expression of SIRPα in the Burn/CLP group suggests a decreased phagocytic capacity and may reflect a compromised immune response.

Recently, Siglec-F has been recognized as a surface marker of neutrophils, in addition to its well-established role as an eosinophil marker. Siglec-F-positive neutrophils were previously detected in nasal lavage fluid under inflammatory conditions (49), in myocardial tissue after acute myocardial infarction (50), and in lung cancer tissue (51, 52). This neutrophil subpopulation has thus been considered to promote tumorigenesis (52). Siglec-F-positive neutrophils have been reported to arise from conventional Siglec-F-negative neutrophils under the influence of TGF-β1 and/or granulocyte-macrophage colony-stimulating factor (GM-CSF) released from damaged tissues (53). Siglec-F-positive neutrophils are rarely detected in the bone marrow, spleen, or peripheral blood, with many reports of their presence in inflamed tissues. Siglec-F-positive neutrophils are known to be hyper-segmented neutrophils and can survive for several days, as opposed to conventional neutrophils, which survive for up to several hours (51). Previous studies have also shown that Siglec-F-positive neutrophils have an enhanced ability to produce ROS and form NETs when compared to Siglec-F-negative neutrophils (49, 53). Although there have been various reports on Siglec-F-positive neutrophils, their characteristics have not yet been fully elucidated, and the significance of their abundance in ascitic fluid of the Burn/CLP group remains elusive.

CD68 is often used as a marker for monocytes or macrophages, but CD68-positive cells from other cell types are also known (54). A previous report described CD68-positive neutrophils in the colon mucosal tissue of patients with inflammatory bowel disease (55). The inflammatory stimuli-induced upregulation of CD68 on macrophages has also been reported (56). Macrophages in CD68-knockout mice have an equivalent phagocytic capacity (57). Thus, the details of the role of CD68 on macrophages/monocytes in immune function have not been clarified, and the same has not been clarified for CD68 on neutrophils. We indicated that CD68-high neutrophils were deficient in the Burn/CLP group, but further study is needed to interpret these results with regard to neutrophil immunocompetence.

## Conclusion

In the present study, we characterized the histological and immunological findings in a murine model of post-traumatic sepsis. We noted apparent changes in the expression of CD11b, SIRPα, Siglec F, and CD68 on neutrophils in model mice. Our results suggest that elevated serum TGF-β1 levels may influence subsequent cytokine suppression and neutrophil phenotype in the pathogenesis of post-traumatic sepsis, facilitating an immunosuppressive state. The functional modulation of neutrophils may represent a potential therapeutic target for the post-traumatic immunosuppressed state in sepsis.

## Data availability statement

The raw data supporting the conclusions of this article will be made available by the authors, without undue reservation.

## Ethics statement

This animal study was reviewed and approved by The Hokkaido University Animal Experiment Regulations and the Institutional Ethical Review Board at Hokkaido University (Approval number: 19-0167).

## Author contributions

AM contributed to the experimentation, analysis, and manuscript preparation. TW contributed to the research concept and oversaw the study. TT contributed to the performance of experiments. YO and ST contributed to the provision of histopathological photographs and pathological evaluation. KK and KY contributed to the immunological evaluation through sample measurements. KY contributed to the revision of the manuscript. All authors read and approved the final version of the manuscript prior to submission.
