# Supplementary material for: Neutrophil phenotypes implicated in the pathophysiology of post-traumatic sepsis
Source: Front Med (Lausanne). 2022 Dec 2;9:982399. doi: 10.3389/fmed.2022.982399 (PMC9757139; doi:10.3389/fmed.2022.982399)
Supplement: Supplementary file 1 [file Data_Sheet_1.docx]

Supplementary Material

1. **Supplementary Figures and Tables**
   1. **Supplementary Figures**


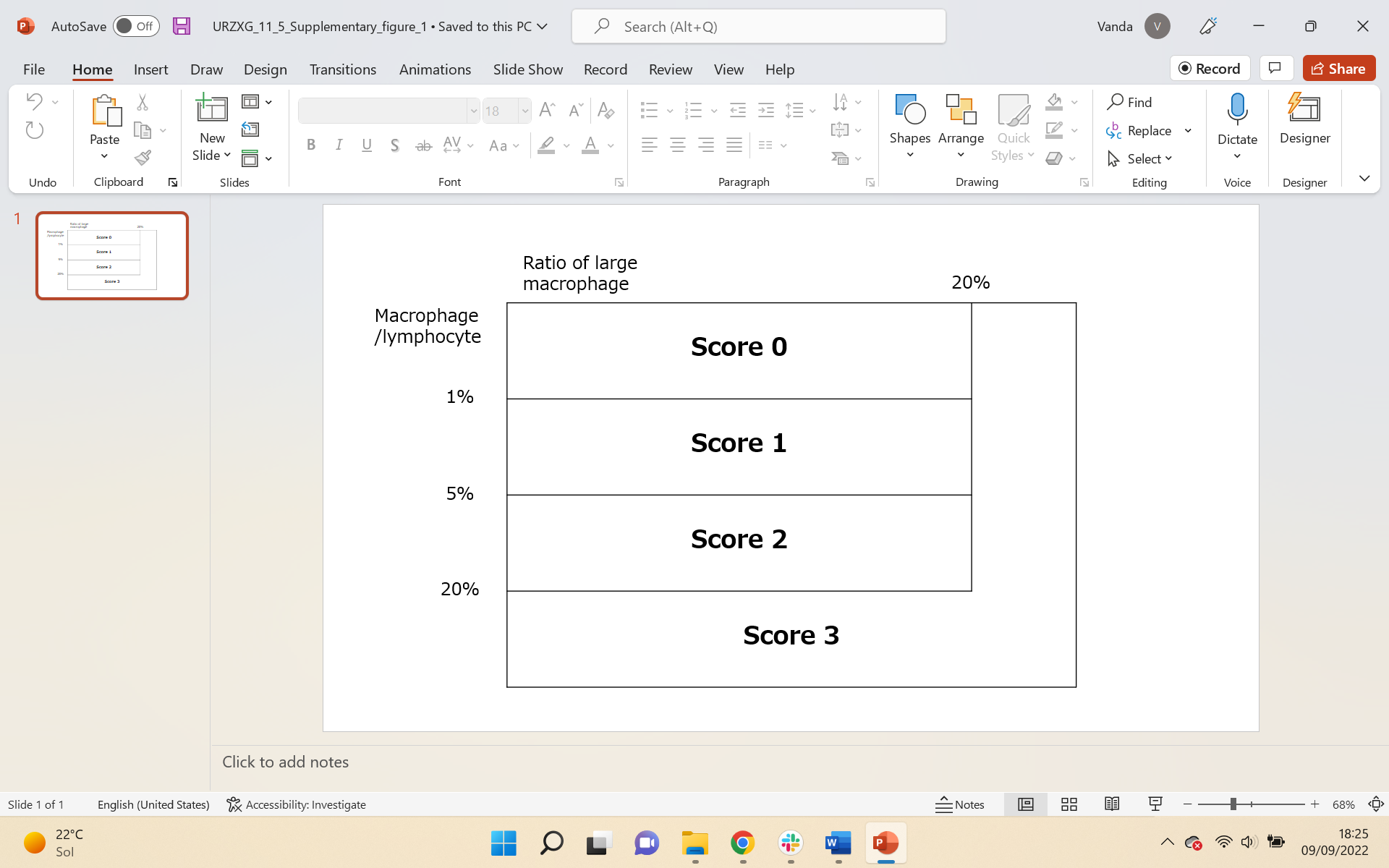


**Supplementary Figure 1**. The scoring system of macrophage infiltration into the spleen.


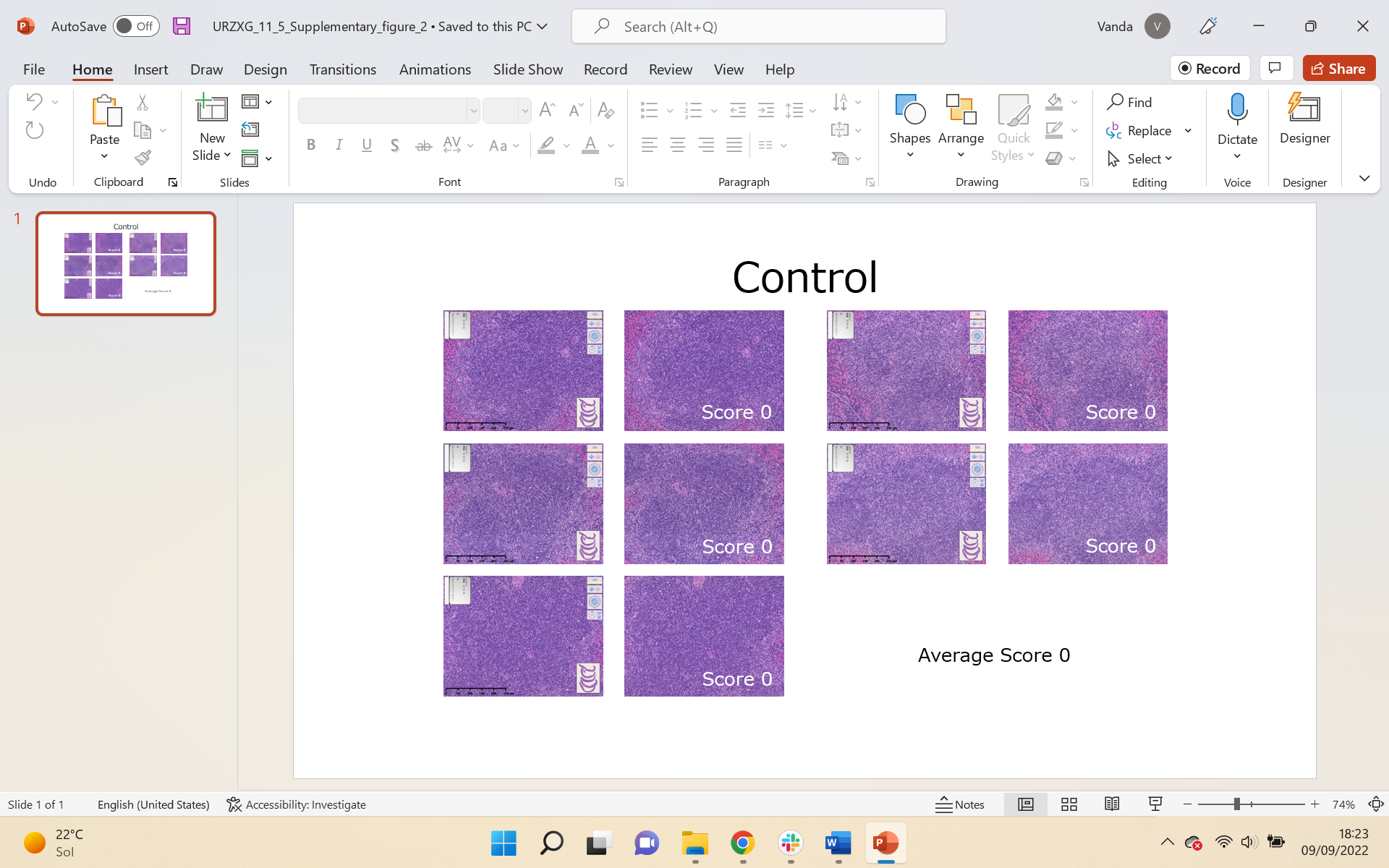


**Supplementary Figure 2**. Spleen tissue and macrophage infiltration scores of control mice.


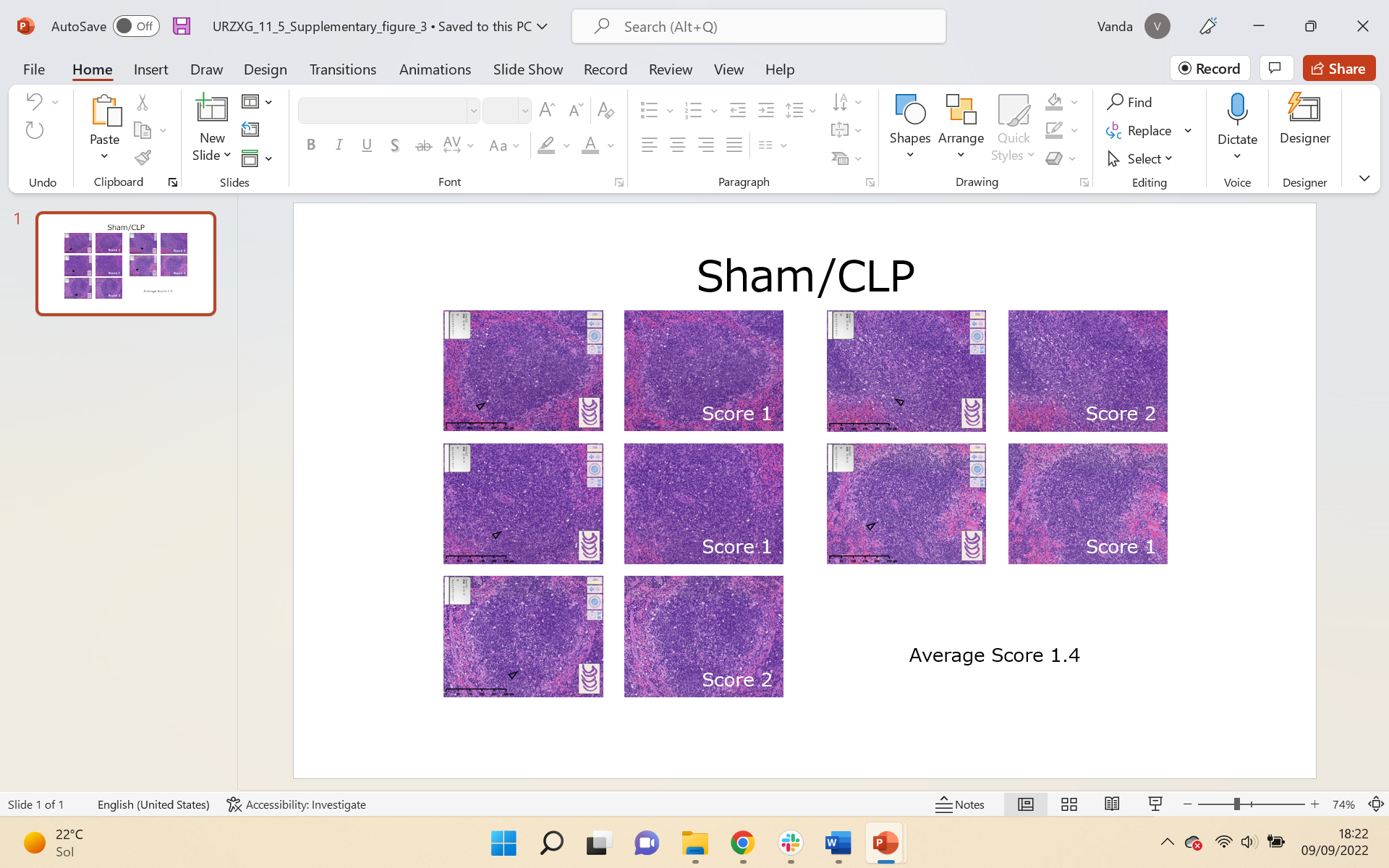


**Supplementary figure 3**. Spleen tissue and macrophage infiltration scores of Sham/CLP mice.


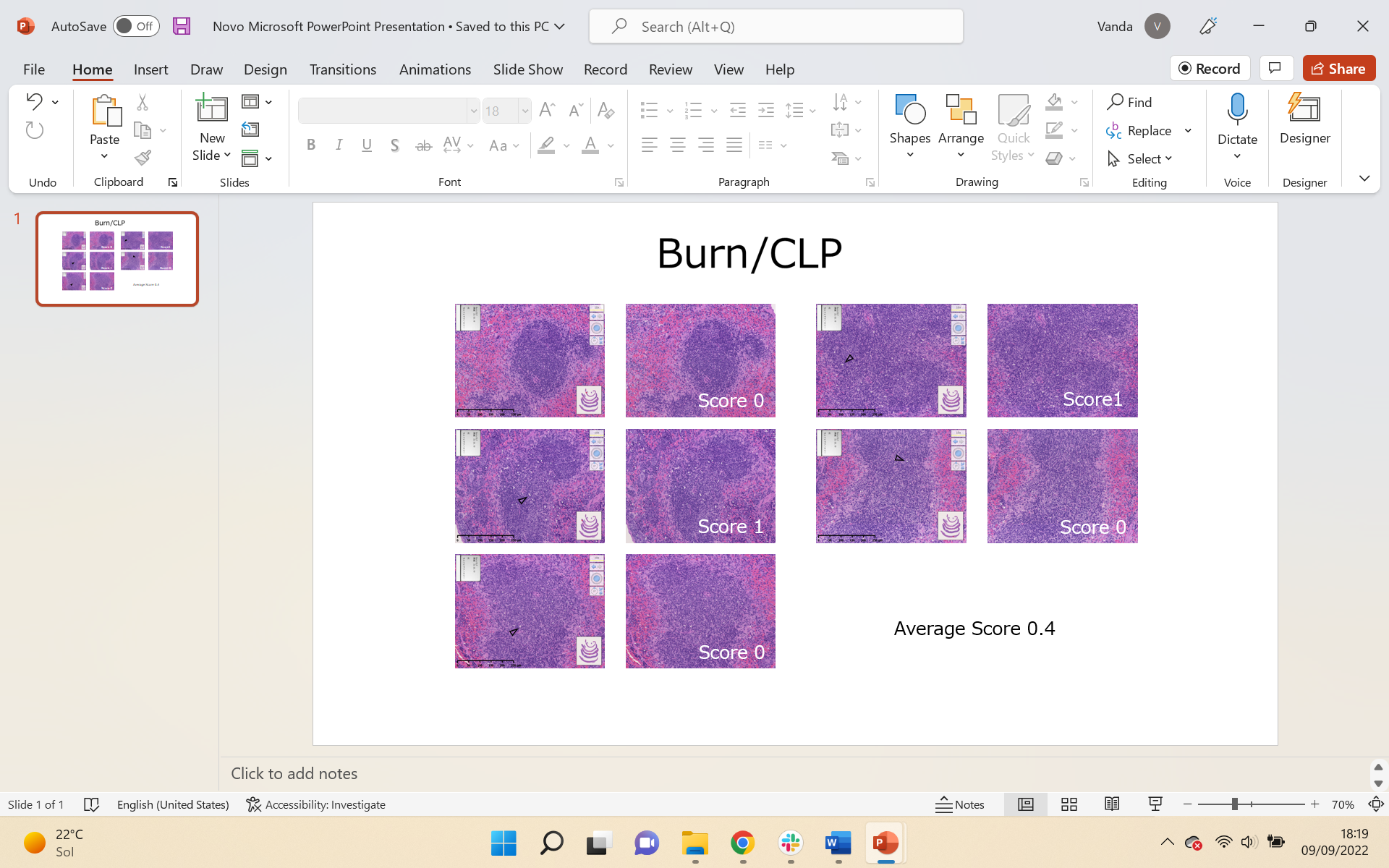


**Supplementary figure 4**. Spleen tissue and macrophage infiltration scores of Burn/CLP mice.

**
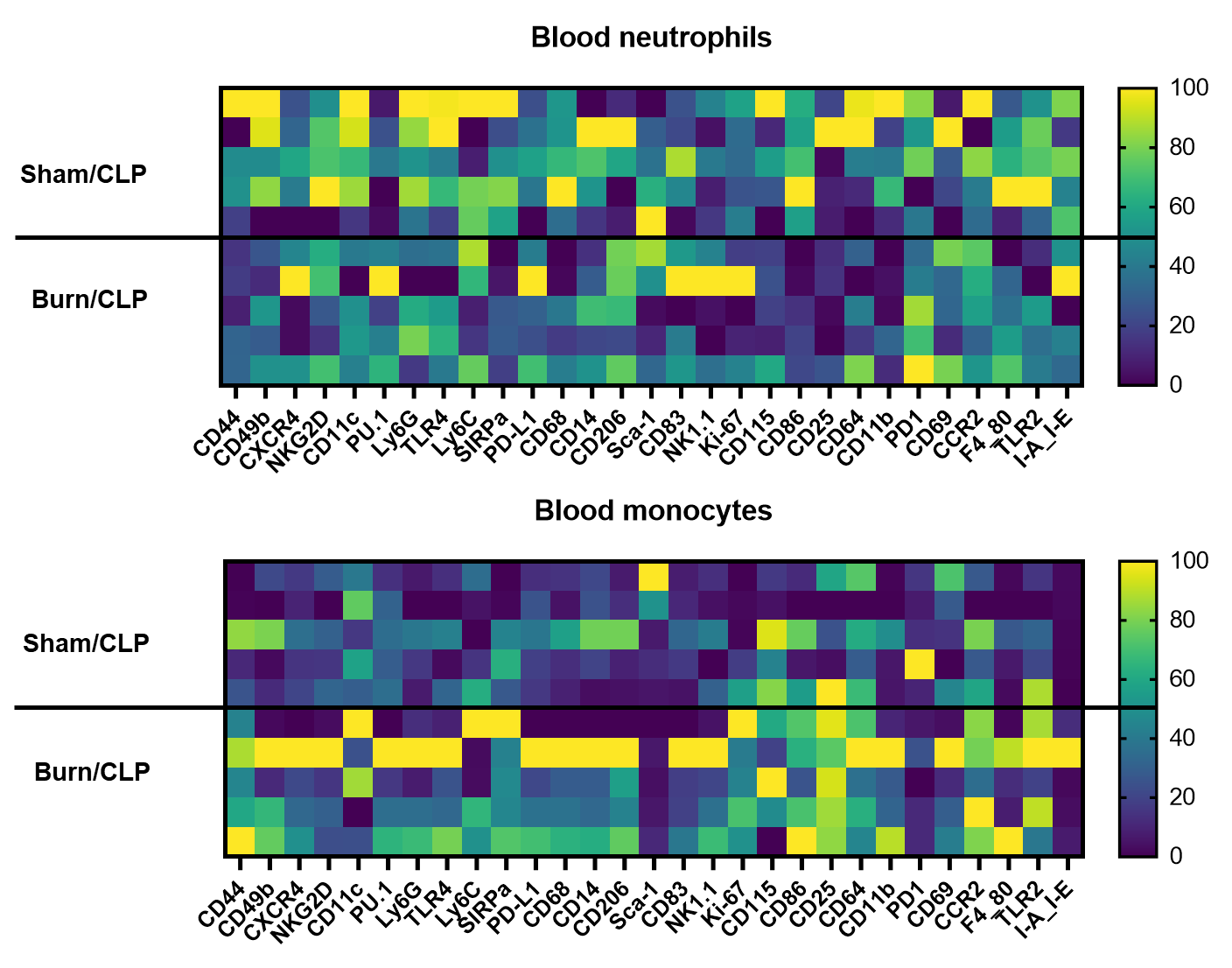
**

**Supplementary figure 5**. Heatmaps of marker expression levels in blood. CCR2, C-C chemokine receptor type 2; CD, cluster of differentiation; CLP, cecal ligation and puncture; CXCR4, C-X-C chemokine receptor type 4; FoxP3, forkhead box P3; Ly6, lymphocyte antigen 6; NKG2D, natural killer group 2D; PD-1, programmed cell death-1; PD-L1, programmed cell death-ligand 1; RORγT, retinoic acid receptor-related orphan receptor gamma t; Sca-1, stem cell antigen-1; SIRPα, signal-regulatory protein alpha; T-bet, T-box expressed in T cells; TCRγδ, T cell receptor gamma delta; TLR4, Toll-like receptor 4.


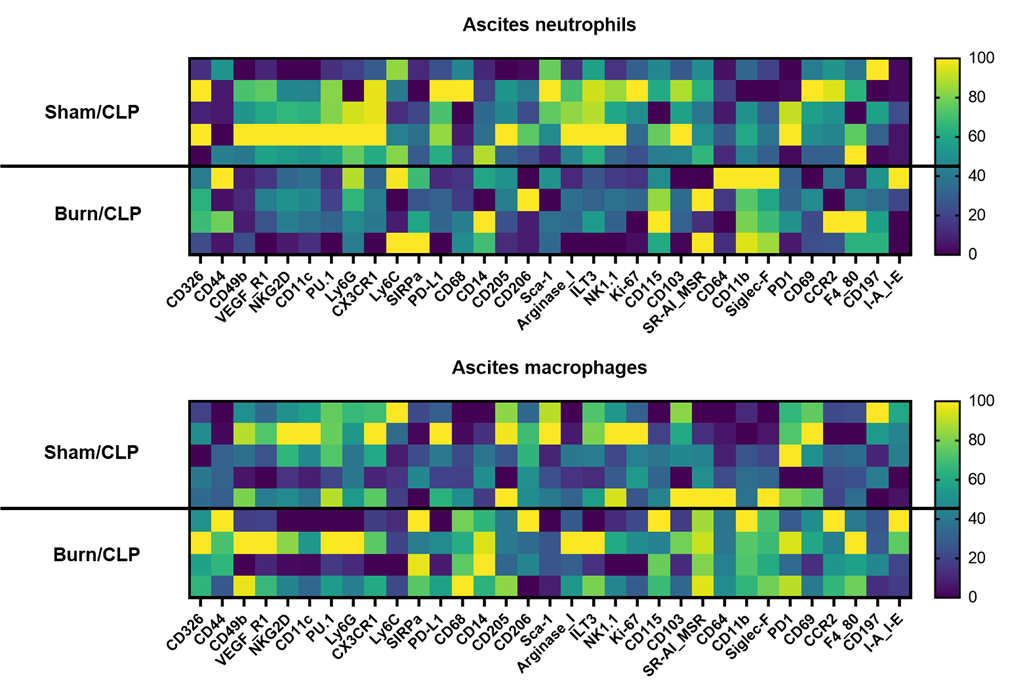


**Supplementary Figure 6**. Heatmaps of marker expression levels in ascitic fluid. CCR2, C-C chemokine receptor type 2; CD, cluster of differentiation; CLP, cecal ligation and puncture; CX3CR1, C-X-3C chemokine receptor type 1; FoxP3, forkhead box P3; ILT3, immunoglobulin-like transcript 3; Ly6, lymphocyte antigen 6; NKG2D, natural killer group 2D; PD-1, programmed cell death-1; PD-L1, programmed cell death-ligand 1; RORγT, retinoic acid receptor-related orphan receptor gamma t; Sca-1, stem cell antigen-1; Siglec-F, sialic acid-binding lg-like lectin F; SIRPα, signal-regulatory protein alpha; SR-AI_MSR, scavenger receptors AI_macrophage scavenger receptor; T-bet, T-box expressed in T cells; TCRγδ, T cell receptor gamma delta; TLR4, Toll-like receptor 4; VEGF_R1, vascular endothelial growth factor receptor 1.

**
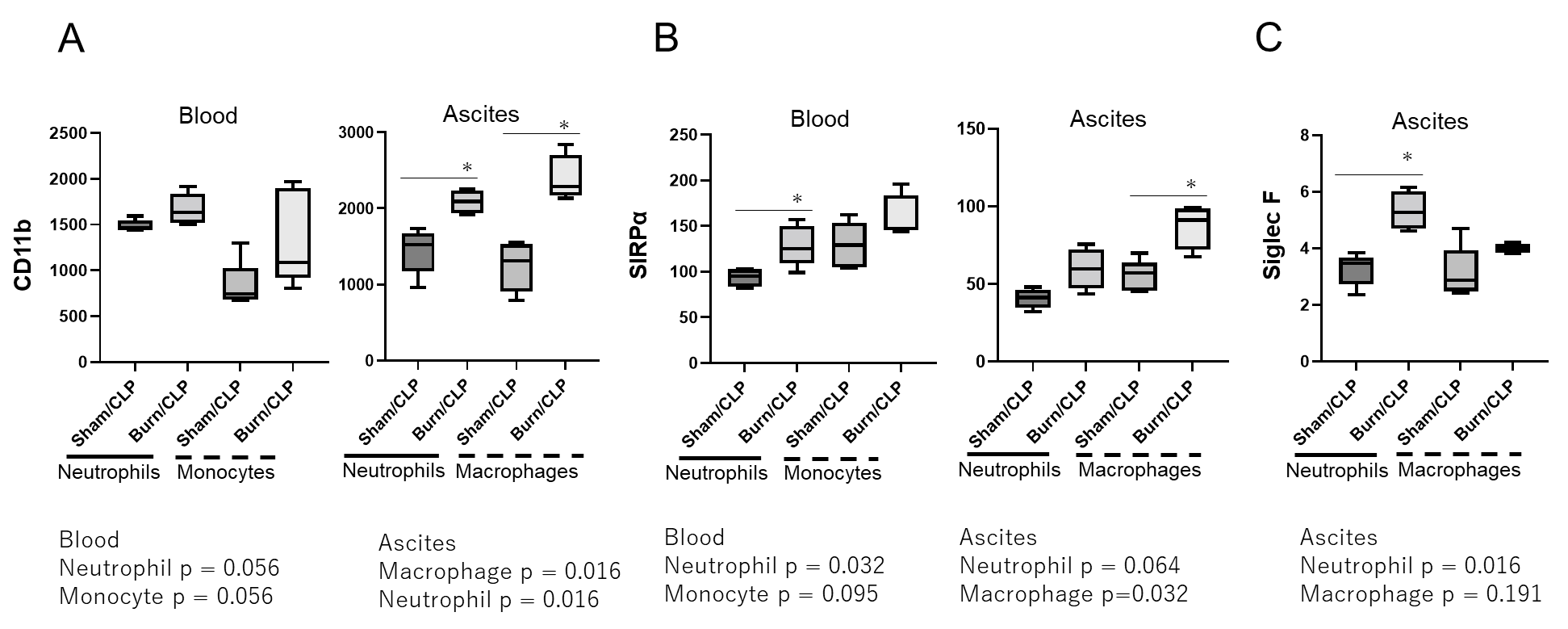
**

**Supplementary Figure 7**. The expressions of CD11b, SIRPα, and Siglec-F on neutrophils and monocytes/macrophages. (A) Expression of CD11b. (B) Expression of SIRPα. (C) Expression of Siglec-F. CD, cluster of differentiation; CLP, cecal ligation and puncture; Siglec-F, sialic acid-binding lg-like lectin F; SIRPα, signal-regulatory protein alpha. ^*^*p* < 0.05.

- 1. **Supplementary Tables**

**Supplementary Table 1**. CyTOF panel of the blood samples.

| **Marker** | **Clone** | **Isotope Label** |  | **Marker** | **Clone** | **Isotope Label** |
| --- | --- | --- | --- | --- | --- | --- |
| **CD45** | 30-F11 | ^89^Y |  | **CD206*** | C068C2 | ^159^Tb |
| **CD4** | RM4-5 | ^115^In |  | **Ly6A/E** | E13-161.7 | ^160^Gd |
| **CD44** | IM7 | ^141^Pr |  | **CD83** | Michel-19 | ^161^Dy |
| **CD8a** | 53-6.7 | ^142Nd^ |  | **FoxP3*** | FJK-16s | ^162^Dy |
| **CD49b** | DX5 | ^143^Nd |  | **NK1.1** | PK136 | ^163^Dy |
| **CD184** | L276F12 | ^144^Nd |  | **Ki-67*** | 8D5 | ^164^Dy |
| **NKG2D** | REA1175 | ^145^Nd |  | **CD115** | REA827 | ^165^Ho |
| **CD11c** | N418 | ^146^Nd |  | **CD86** | GL-1 | ^166^Er |
| **PU.1*** | phpu13 | ^147^Sm |  | **CD25** | 3C7 | ^167^Er |
| **Ly6G** | 1A8 | ^148^Nd |  | **CD64** | x54-5/7.1 | ^168^Er |
| **CD19** | 6D5 | ^149^Sm |  | **CD11b** | M1/70 | ^169^Tm |
| **TLR4** | MTS510 | ^150^Nd |  | **RORγt*** | REA278 | ^170^Er |
| **Ly6C** | HK1.4 | ^151^Eu |  | **CD279** | 29F.1A12 | ^171^Yb |
| **CD3e** | 145-2C11 | ^152^Sm |  | **TCRγδ** | GL3 | ^172^Yb |
| **CD172a** | P84 | ^153^Eu |  | **CD69** | 310106 | ^173^Yb |
| **CD274*** | 10F.9G2 | ^154^Sm |  | **CCR2** | 475301R | ^174^Yb |
| **CD68*** | FA-11 | ^155^Gd |  | **F4/80** | T45-2342 | ^175^Lu |
| **CD14** | Sa14-2 | ^156^Gd |  | **TLR2*** | T2.5 | ^176^Yb |
| **T-bet*** | 4B10 | ^158^Gd |  | **I-A/I-E** | M5/114.15.2 | ^209^Bi |

*Intracellular marker. CCR2, C-C chemokine receptor type 2; CD, cluster of differentiation; CyTOF, cytometry by time-of-flight; FoxP3, forkhead box P3; Ly6, lymphocyte antigen 6; NKG2D, natural killer group 2D; RORγT, retinoic acid receptor-related orphan receptor gamma t; T-bet, T-box expressed in T cells; TCRγδ, T cell receptor gamma delta; TLR4, Toll-like receptor 4.

**Supplementary Table 2.** CyTOF panel of ascites fluid samples.

| **Marker** | **Clone** | **Isotope Label** |  | **Marker** | **Clone** | **Isotope Label** |
| --- | --- | --- | --- | --- | --- | --- |
| **CD45** | 30-F11 | ^89^Y |  | **CD206*** | C068C2 | ^159^Tb |
| **CD326** | G8.8 | ^113^In |  | **Ly6A/E** | E13-161.7 | ^160^Gd |
| **CD4** | RM4-5 | ^115^In |  | **Arginase I*** | Polyclonal | ^161^Dy |
| **CD44** | IM7 | ^141^Pr |  | **CD85k** | H1.1 | ^162^Dy |
| **CD8a** | 53-6.7 | ^142^Nd |  | **NK1.1** | PK136 | ^163^Dy |
| **CD49b** | DX5 | ^143^Nd |  | **Ki-67*** | 8D5 | ^164^Dy |
| **VEGF R1** | 141522 | ^144^Nd |  | **CD115** | REA827 | ^165^Ho |
| **NKG2D** | REA1175 | ^145^Nd |  | **CD103** | 2E7 | ^166^Er |
| **CD11c** | N418 | ^146^Nd |  | **SR-AI** | 268318 | ^167^Er |
| **PU.1*** | phpu13 | ^147^Sm |  | **CD64** | x54-5/7.1 | ^168^Er |
| **Ly6G** | 1A8 | ^148^Nd |  | **CD11b** | M1/70 | ^169^Tm |
| **CD19** | 6D5 | ^149^Sm |  | **Siglec F** | E50-2440 | ^170^Er |
| **CX3CR1** | SA011F11 | ^150^Nd |  | **CD279** | 29F.1A12 | ^171^Yb |
| **Ly6C** | HK1.4 | ^151^Eu |  | **TCRγδ** | GL3 | ^172^Yb |
| **CD3e** | 145-2C11 | ^152^Sm |  | **CD69** | 310106 | ^173^Yb |
| **CD172a** | P84 | ^153^Eu |  | **CCR2** | 475301R | ^174^Yb |
| **CD274** | 10F.9G2 | ^154^Sm |  | **F4/80** | T45-2342 | ^175^Lu |
| **CD68*** | FA-11 | ^155^Gd |  | **CD197** | REA685 | ^176^Yb |
| **CD14** | Sa14-2 | ^156^Gd |  | **I-A/I-E** | M5/114.15.2 | ^209^Bi |
| **CD205** | NLDC-145 | ^158^Tb |  |  |  |  |

*Intracellular marker. CCR2, C-C chemokine receptor type 2; CD, cluster of differentiation; CyTOF, cytometry by time-of-flight; CX3CR1, C-X-3C chemokine receptor 1; Ly6, lymphocyte antigen 6; NKG2D, natural killer group 2D; RORγT, retinoic acid receptor-related orphan receptor gamma t; Siglec F, sialic acid-binding lg-like lectin F; SR-AI, scavenger receptor-AI; T-bet, T-box expressed in T cells; TCRγδ, T cell receptor gamma delta; TLR4, Toll-like receptor 4; VEGF R1, vascular endothelial growth factor receptor 1.
